# Modified Buzhong Yiqi decoction for myasthenia gravis: A systematic review protocol

**DOI:** 10.1097/MD.0000000000013677

**Published:** 2018-12-14

**Authors:** Xiaotao Jiang, Guoming Chen, Jiahua Huang, Linling Xie, Danting Shen, Kailin Jiang, Hua Xu

**Affiliations:** a Guangzhou University of Chinese Medicine; b Department of Paediatrics, First Affiliated Hospital of Guangzhou University of Chinese Medicine, Guangzhou, China.

**Keywords:** Buzhong Yiqi decoction, myasthenia gravis, protocol, systematic review

## Abstract

**Background:**

Myasthenia gravis (MG) is an autoimmune disease caused by the transmission of dysfunction in the neuromuscular junction, manifesting partial or systemic skeletal muscle weakness and fatigue, which are exacerbated by activities and relieved after rest. Currently, the conventional therapy is applying cholinesterase inhibitors, steroids, immunosuppressant, and thymectomy. However, these drugs have obvious side effects. According to traditional Chinese medicine (TCM) theory, Buzhong Yiqi decoction (BYD) is a Qi-supplementing formula which is suitable for MG management as MG is generally diagnosed as “flaccidity syndrome” and considered caused by Qi-deficiency. An increasing number of clinical controlled studies also have found that BYD could improve the efficacy and reduced adverse effects in treating MG, but there is no systematic review of it. Therefore, we will use meta-analysis to evaluate the efficacy and safety of BYD for MG.

**Methods:**

PubMed, MEDLINE, EMBASE, Cochrane Library, China National Knowledge Infrastructure (CNKI), Wanfang data, Chinese Scientific Journals Database (VIP), and China biomedical literature database (CBM) will be searched to obtain the eligible studies published up to June 1, 2018. The primary outcome will be clinical absolute score before and after treatment, clinical relative score as well as effective rate. The secondary outcome will be the concentration of acetylcholine receptor antibody (AchRAb) in serum and adverse events incidence. Data analysis will be conducted using RevMan5.3 and Stata V.9.0 software. Trial sequential analysis (TSA) will be performed to assess the risk of random error and the validity of conclusion using TSA program version 0.9 beta.

**Results:**

This systematic review will provide a high-quality synthesis of BYD and its modified forms for MG from various evaluation aspects including clinical absolute score before and after treatment, clinical relative score, effective rate, the concentration of AchRAb in serum and adverse events incidence.

**Conclusion:**

The systematic review will provide evidence to assess the efficacy and safety of BYD and its modified forms in the treatment of MG.

**Prospero registration number:**

PROSPERO CRD42018095241.

## 1 Introduction

Myasthenia gravis (MG) is an autoimmune disease caused by dysfunctions of transmission in the neuromuscular junction.^[1]^ The major clinical manifestations are partial or systemic skeletal muscle weakness and fatigue, which exacerbated by activities and across the day while improved after rest.^[2]^ It was reported that the incidence of MG was around 30/1,000,000 per year^[2]^ with approximately 40% mortality.^[3]^ Currently, the commonly used therapeutic strategies are cholinesterase inhibitors, steroids, immunosuppressant, and thymectomy, which are effective to control the symptoms and reduce the mortality. However, long-term use of steroids or immunosuppressant may induce osteoporosis, immunosuppression and other adverse effects.^[4,5]^

According to traditional Chinese medicine (TCM) theory, MG is generally diagnosed as “flaccidity syndrome” and “Qi-deficiency pattern”, which means that Qi-supplementing strategy is vital in MG management. Buzhong Yiqi decoction (BYD), a Qi-supplementing formula in TCM, has demonstrated better efficacy and fewer adverse effects when combining with conventional therapy on MG in increasing number of clinical controlled studies. In a study, BYD up-regulates the expression of phosphorylated myosin light chain in rats which promoted myocutaneous movement and improved muscle contractility.^[6]^ In another rats study, BYD could relieve MG symptoms with a decreased level of anti-R97-116 IgG1 and the down-regulation of TNF-α.^[7]^ These may be the mechanism of BYD in treating MG.

Management of MG with BYD is common in China, but there is no systematic review of it. Therefore, based on the extensive collection of literature, we will use meta-analysis to evaluate the efficacy and safety of BYD and its modified forms in the treatment of MG.

## 2 Methods

### 2.1 Study registration

We have registered the protocol in the International Prospective Register of Systematic Reviews (PROSPERO) with registration number CRD42018095241 on May 23, 2018.

### 2.2 Ethics and dissemination

Individual data of each patient will not be needed in our research as this is a systematic review. Therefore an ethics committee or institutional review board approval is not needed. Our aim is to publish the results in a peer-reviewed journal. The results of this review will provide information about the safety and efficacy of BYD and its modified forms in the treatment of MG, therefore, helping clinicians make decisions on clinical practice.

### 2.3 Types of studies

All RCTs comparing the effect and safety of BYD on MG will be pooled, regardless of the period of treatment and preparation.

### 2.4 Participants

Patients diagnosed based on China Guidelines for the Diagnosis and Treatment of MG^[8]^ will be included.

The diagnosis criteria contain following items:

- Clinical manifestation: Some specific striated muscle with patchy distribution is weakness and display volatility as well as ease of fatigue. The symptoms will be exacerbated by activities and relieved after rest. Ocular muscle involvement is most common.
- Pharmacological characteristics: Positive outcome of neostigmine test.
- Neuroelectrophysiological characteristics: In repetitive nerve stimulation (RNS), the amplitude of compound muscle action potential (CMAP) decreases more than 10%. In single fiber EMG (SFEMG), 2 or more Jitter widen (> 55 ms) with or without blocks.
- Serological characteristics: Anti-AChR is detected in blood sample. Anti-MuSK and LRP-4 are detectable in very few patients with MG.

Clinically, on the basis of clinical manifestations with positive pharmacological or electrophysiological outcomes, it can be diagnosed as MG. Detection of anti-AChR or other related antibodies in blood sample helps to further confirm the diagnosis. Excluding other diseases is necessary.

### 2.5 Types of interventions

The patients in the treatment groups will be given BYD or modified BYD using as a monotherapy or in combination with conventional therapy. BYD consisting of 8 herbs: Radix atragali, Radix codonopsis, Prepared Radix Glycyrrhizae, Radix Angelicae sinensis, Pericarpium citri reticulatae, Rhizoma cimicifugae, Radix bupleuri, and Rhizoma atractylodis macrocephalae. According to “Jun-Chen-Zuo-Shi” principle of Chinese herbal formula, Radix atragali is “Jun” herb and Radix codonopsis, Prepared Radix Glycyrrhizae as well as Rhizoma atractylodis macrocephalae are “Chen” herb, both of which are the core of BYD. Therefore, modified BYD should include Radix atragali, Radix codonopsis, Prepared Radix Glycyrrhizae, and Rhizoma atractylodis macrocephalae basically. The number of modified herbs will not exceed 5 (n ≤5). The preparation, dosage and period will not be considered. Patients of control group will be treated with conventional therapy in accordance with experiment group such as cholinesterase inhibitors, steroids, immunosuppressant, and so on.

### 2.6 Outcome measures

#### 2.6.1 Primary outcomes

- Clinical absolute score before and after treatment: The clinical absolute score based on The Chinese Expert Consensus on The Diagnosis and Treatment of MG will be calculated according to the score of following items: upper eyelid weakness, upper eyelid fatigue test, limitation of eyeball horizontal movement, upper and lower limb fatigue test, the facial muscle weakness, the function of chewing, swallowing, and respiratory muscle. Clinical absolute score will be calculated before and after treatment.
- Clinical relative score and effective rate: The clinical relative score = (absolute score before treatment—absolute score after treatment)/absolute score before treatment × 100%. Effective rate will be assessed according to the clinical relative score. Clinical relative score ≥80% is cured, 50% to 79% is markedly improved, 25% to 49% is improved, and <25% is invalid. N1, N2, N3, and N are the number of patients who are cured, markedly improved, improved, and the sample size respectively, which will be used to calculate the effective rate (effective rate = N1+N2+N3/N). This evaluation method is based on the Chinese Expert Consensus on Diagnosis and Treatment of MG.^[9]^

#### 2.6.2 Secondary outcomes

- Acetylcholine receptor antibody (AchRAb): The concentration of AchRAb in Serum.
- Adverse events incidence.

### 2.7 Search strategy

Following databases will be searched: PubMed, MEDLINE, EMBASE, Cochrane Library, China National Knowledge Infrastructure (CNKI), Wanfang data, Chinese Scientific Journals Database (VIP), and China biomedical literature database (CBM). We will select the eligible studies published up to June 1, 2018. Medical Subject Headings and non-MeSH terms will be used to search the studies in various combinations. The following terms including: “Buzhong Yiqi”, “BuzhongYiqi”, “myasthenia gravis”, “myasthenia”, “flaccidity”, “paralysis”, “Weizheng”, will be searched individually or in combination. We will not apply any language, population or country restrictions.

The specific search strategy will be (taking PubMed as an example):

- #1 MG [MeSH] or myasthenia [Title/Abstract] or flaccidity [Title/Abstract] or paralysis [Title/Abstract] or Weizheng[Title/Abstract]
- #2 Buzhong Yiqi [MeSH] or BuzhongYiqi [Title/Abstract]
- #3 #1 and #2.

The strategy will be modified for other databases use if necessary. The reference lists of the relevant articles will also be checked

### 2.8 Data collection and analysis

#### 2.8.1 Study selection

We will select RCTs comparing the effect and safety of BYD on MG. Those articles meeting 1 of following items will be excluded:

(i) the duplicates,
(ii) the participants did not meet the diagnosis criteria of MG or the diagnosis criteria is unknown,
(iii) not RCT studies,
(iv) the studies in which the experimental participants do not receive modified BYD as a monotherapy or in combination with conventional therapy as the primary intervention,
(v) the intervention contains any other TCM therapy, and
(vi) incomplete data which will be required.

**Figure 1:**
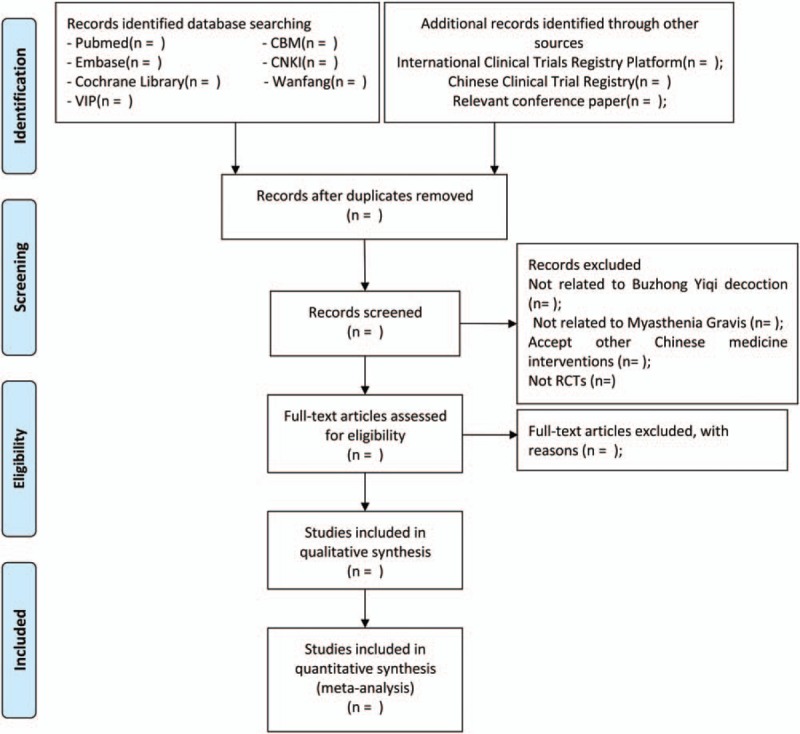
Flow diagram of study selection process.

Two reviewers (XL and SD) will evaluate whether the studies are eligible. A third reviewer (HJ) will be required to discuss when there is any disagreement during the article's inclusion. A Preferred Reporting Items for Systematic Reviews and Meta-Analyses (PRISMA) flow diagram will be used to exhibit the specific process of studies screening (Fig. 1).

#### 2.8.2 Data extraction

Data will be independently extracted according to predefined criteria by 2 reviewers (XL and SD). When there is any disagreement, all reviewers will discuss to solve it. The following items will be extracted: the first author, the year of publication, type of MG, duration, the patient characteristics, the sample size of each study, interventions, clinical absolute score before and after treatment, clinical relative score, effective rate, the concentration of AchRAb in serum, and adverse events.

### 2.9 Risk of bias assessment

Two reviewers (XL and SD) will independently appraise the risk of bias of each included article according to the Cochrane Handbook for Systematic Reviews of Interventions. The methodological quality will be evaluated from the following 7 aspects: random sequence generation, allocation concealment, blinding of participants and personnel, blinding of outcome assessments, incomplete outcome data, selective reporting, and other bias. Description of each aspect of risk of bias in each pooled study will be conducted in order to provide the rationale for the risk judgment. The risks will be categorized as low, high, or unclear with a graphical presentation.

### 2.10 Data synthesis

RevMan 5.3 software will be used to analyze the related research indicators. Effective rate will be expressed by risk ratios (RR) with 95% confidence intervals (CIs), while adverse events incidence expressed by odds ratio (OR) with 95% CIs. Clinical absolute score before and after treatment, clinical relative score, and the concentration of AchRAb in serum will be expressed by weighted mean difference (WMD) with 95% CIs. Heterogeneity between the studies will be evaluated using the I-squared statistic. I^2^ <50% is considered no heterogeneity. I^2^ >50% indicates a significant heterogeneity, and under this circumstance, sensitive analysis, meta-regression analysis as well as subgroup analysis based on MG subtype, period, age, level of risk of bias, and etc will be performed. The choice of random effect model or fixed effect model will depend on whether there exists heterogeneity or not. If the extracted data are insufficient quantitative, we will conduct qualitative synthesis of them.

### 2.11 Assessment of reporting biases

Egger or Begg test will be conducted to analyze the potential publication bias when the amount of pooled studies is sufficient (n ≥10). If publication bias exists, we will use shearing and mending method to assess the impact of publication bias on the results.

### 2.12 Trial sequential analysis (TSA)

To solve the problem about the repeated significance test on cumulative data increasing the overall risk of I errors in a single RCT, statistical monitoring boundaries will be adopted to assess if a single test could reach an in-advance termination with a *P* value small enough to show the expected effect or futility.^[10,11]^ Through combining information size estimation with an adjusted threshold for statistical significance in the cumulative meta-analysis, TSA is always used to assess the risk of I errors.^[10,12]^ Trial sequential monitoring boundaries calculated using Obrien–Fleming alpha spending function adjusts the CIs and reduce type I errors. The reliability of expected intervention efficacy would be evidenced if the trial sequential monitoring boundaries are crossed by the cumulative z curve, and under this circumstance, no further trials should be conducted.^[13]^ Without the crossing of the boundaries and required information size, an stable conclusion cannot be drawn.^[14]^ In our assumption, type I error will be α = 0.05 and type II error will be set at β = 0.20. Relative risk reduction and the incidence in control arm will be derived from the meta-analysis outcomes. We will use TSA 0.9 (Copenhagen Trial Unit, Copenhagen, Denmark) to perform TSA to assess the risk of random error and the validity of conclusion.

### 2.13 Quality of evidence

We will adopt The Grading of Recommendations Assessment, Development, and Evaluation approach to assess the quality of evidence of the pooled studies. Limitations of the study, inconsistencies, indirect evidence, inaccuracies, and publication bias will be considered. Levels of evidence quality will be classified into 4 levels: high, moderate, low, or very low.

## 3 Discussion

MG resulted from the binding of autoantibodies to proteins involved in signaling at the neuromuscular junction, is an autoimmune syndrome caused by the failure of neuromuscular transmission.^[15]^ Cholinesterase inhibitors are the first-line drug to relieve symptoms in general MG while immunosuppression with steroids, azathioprine, ciclosporin, tacrolimus, and etc are applied as definitive therapy. However, long-term use of them always causes severe adverse effects.^[16]^ According to TCM theory, BYD is applicable for MG as BYD is a Qi-supplementing formula and properly matching with the Qi-deficiency syndrome of MG. Numerous researches also have demonstrated that BYD could improve the efficacy and lower the total number of adverse effects in treating MG. To our knowledge, there is no related systematic review published in English. Therefore, we conduct this systematic review to further evaluate the effectiveness and safety of BYD for MG. Our aim is to provide more clinical evidence helping clinicians make decisions on clinical practice in MG treatment.

## Author contributions

Xiaotao Jiang, Guoming Chen and Hua Xu designed the study. Xiaotao Jiang, Guoming Chen drafted the protocol. All authors revised the manuscript. All authors approved the final version.

**Conceptualization:** Xiaotao Jiang, Guoming Chen.

**Methodology:** Xiaotao Jiang, Hua Xu.

**Writing-original draft:** Xiaotao Jiang, Guoming Chen.

**Writing-review & editing:** Xiaotao Jiang, Guoming Chen, Jiahua Huang, Linling Xie, Danting Shen, and Kailin Jiang.

**Supervision:** Hua Xu.

**Conceptualization:** Xiaotao Jiang, Guoming Chen, Hua Xu.

**Methodology:** Xiaotao Jiang, Hua Xu.

**Supervision:** Hua Xu.

**Writing – original draft:** Xiaotao Jiang, Guoming Chen.

**Writing – review & editing:** Xiaotao Jiang, Guoming Chen, Jiahua Huang, Linling Xie, Danting Shen, Kailin Jiang.

Hua Xu orcid: 0000-0003-2212-7347.
